# Costs and Savings Associated With the Police Use of the interRAI Brief Mental Health Screener

**DOI:** 10.3389/fpsyt.2021.726469

**Published:** 2021-10-18

**Authors:** Ron Hoffman, Jeffrey Harman, Heidi Kinsell, Gregory Brown

**Affiliations:** ^1^School of Criminology and Criminal Justice, Nipissing University, North Bay, ON, Canada; ^2^Department of Behavioral Sciences and Social Medicine, Florida State University College of Medicine, Tallahassee, FL, United States; ^3^Department of Geriatrics, Florida State University College of Medicine, Tallahassee, FL, United States

**Keywords:** costs, resources, policing, mental, disorder

## Abstract

**Background:** The police response to calls for service identified as being related to mental health continues to be highly controversial. Strategies to improve the police response include Crisis Intervention Team (CIT) training and various forms of co-response models neither of which have been subjected to comprehensive evaluations, particularly as to cost-efficiency. A new approach is the use of the interRAI Brief Mental Health Screener to enhance police officer ability to identify persons with serious mental disorders. The purpose of the current study is to evaluate the costs and cost efficiency of the police response to mental health calls using the interRAI Brief Mental Health Screener.

**Method:** Secondary data was analyzed from the use of the screener from 2018 to 2020 by police officers in a mid-sized Canadian city. Changes were measured in the overall number of interactions police officers had with persons with mental health disorders, the number of incidents where police officers referred the person to hospital, and the time officers remained in the emergency department.

**Results:** A total of 6,727 assessments were completed with involuntary referrals decreasing by 30%, and voluntary referrals by 34%. The overall time police officers were involved in involuntary referrals decreased from 123 min in 2018 to 113 min in 2020. The average emergency department wait time for voluntary referrals dropped from 41 min in 2018 to 27 min in 2020, while involuntary referrals decreased from 61 min in 2018 to 42 min in 2020. Each averted involuntary referral to the emergency department resulted in a savings of $81, on average during the study period.

**Conclusion:** An analysis of the costs and costs savings associated with the use of the screener demonstrate that it is a worthwhile investment for police services. An additional benefit is its ability to collect mental health statistics that may be useful to police leaders to justify budgets. Future studies should attempt to devise some method of collecting pre-implementation data that would reveal the true costs and cost-efficiency of using the BMHS, which have been shown to be significant in the current study however, undoubtedly are under-estimated.

## Introduction

During the course of their duties, police officers interact with persons exhibiting a broad range of behaviors related to mental disorder from indicators of disordered thought alone to behaviors that may pose a risk of harm to themselves or to others. Public attention is often drawn to negative incidents such as those involving excessive use of force, and on the question of whether police officers receive sufficient training on mental health issues (1–8). Police officers themselves often feel ill-prepared for calls for service identified as being related to mental health (6, 7). Additionally, police leaders have voiced concern over how such calls are particularly resource-intensive and costly due to the number of officers involved, the overall length of the calls and the time and resources involved in transporting the person to hospital (9–14). Although considerable time, effort and public funds have been directed toward implementing new approaches to improve the overall police response to persons with serious mental disorders (PSMD), evaluating their overall effectiveness and cost-effectiveness has been problematic (15, 16).

Two major new approaches designed to help police better respond to PSMD involve providing additional training for frontline police officers and implementing co-responder models comprised of police officers and mental health professionals (15). One of the most popular models to enhance police training is the Crisis Intervention Team (CIT), where police officers volunteer to receive additional training on mental health issues (17, 18). The Co-Response Team (CRT) model, referred to as the Mobile Crisis Intervention Team (MCIT) in United States, Canada and Australia and “street triage” in the United Kingdom consists of a police officer paired with a mental health professional to respond to calls for service identified as being related to mental health (19–23). While the CIT model continues to be popular in the United States, Canada and Australia (17, 19), CRTs are currently the most prevalent model in the United Kingdom (22).

A third approach to improving the police response to PSMD is the Hub/Situation Table model based on the work of Scotland's Violence Reduction Unit and the Hub model introduced in Saskatchewan, Canada (15). Known as the Community Safety and Well-being movement, proponents of the model believe that calls for service such as those involving mental health, homelessness and substance abuse are largely social issues as opposed to criminal matters and therefore should not be the sole responsibility of police organizations. Instead, the responsibility should be shared with other community organizations that are better equipped to respond. The “Situation Tables” is where representatives of various community agencies meet to discuss the most appropriate intervention for person(s) identified as being at elevated risk (24). This sharing of responsibility would should therefore lead to better outcomes for those experiencing the problems while at the same time easing the demand on police and police resources (24–27). The Hub/Situation Table model continues to expand across Canada where over 100 communities have adopted it (15).

Although these models represent innovative attempts to improve the police response to PSMD, few formal studies exist that have evaluated either their effectiveness or cost effectiveness (15, 22). For example, the goals of CIT are to divert PSMDs away from the criminal justice system, enhance collaboration between police and community organizations, provide accessible mental health services and improve officer and client safety (15). Although the model has had some positive results such as improved officer attitudes and confidence in de-escalating encounters, there is controversy over whether the initial goal of enhancing officer and client safety has been achieved (17, 18, 28). The few studies examining the cost effectiveness of the CIT model suggest modest savings primarily through the reduction in the use of police custody and the number of persons taken to hospital (or place of safety) by police officers (28–30). Similarly, the few studies examining the cost effectiveness of CRT models suggest that police organizations have realized some savings primarily through the reduced time spent on calls and time spent waiting in hospitals (31–35). A meta-analytic study suggests that the CRT model produces a stronger overall impact than crisis intervention training alone however the authors of the study recommend more research is required (36, 37). As to the Hub/Situation Table model, although it continues to expand and mental health cases represent a large proportion of the cases, there are currently no independent, peer-reviewed studies to support its effectiveness or whether its use has resulted in lower costs to police agencies (15).

A more recent attempt at improving the response to PSMD is the interRAI Brief Mental Health Screener (BMHS), an evidence-based instrument used to help police officers identify serious mental health issues according to behaviors indicative of disordered thought and risk of harm to self or others as opposed to symptoms or diagnoses (38–40). Software built around the BMHS enables officers to complete the assessment on their cell phones and to measure, with a series of algorithms, the capacity of the person to care for themselves, and the level of risk of harm they pose to themselves or others (41) which is consistent with police apprehension authorities. For example, in Canada mental health legislation authorizes police officers to take apparently mentally disordered individuals into custody without prior judicial authorization where there is a fear for the life or safety of the person, or a fear they are a risk to cause harm to others (42).

Though no evaluative studies exist, the BMHS has a distinct advantage over other models in that in addition to its potential to improve officers' ability to recognize indicators of serious mental disorder, it can track calls for service identified as being related to mental health. A major impediment to evaluating the effectiveness and cost-effectiveness of the various models designed to improve the police response is the fact that police services typically do not collect data related to non-crime related calls for service such as those involving mental health, homelessness and substance abuse. From a police organization perspective, the ability to monitor police responses to PSMD in real time, and to track the number of officers and time involved in the call supports deployment and efficient use of resources and helps to estimate costs. Thus, the BMHS has the additional capability of providing a financial basis upon which to evaluate the police vs. alternative, diversionary responses, and the most efficient use of resources.

The growing budgetary constraints on police services, the mounting resource costs related to police responses to PSMD is a serious concern, and the cost-effectiveness of CRT, CIT, and Hub/Situation Table models of police response suggest little or no improvement or reduction in use of police resources. The current study represents one of the few research-based efforts to describe and evaluate the police response and use of resources related to calls for service that involve PSMD, making use of data collected with the BMHS in one mid-size police service in Canada.

## Materials and Methods

### Setting

The setting for the study was Guelph, a mid-sized city in southwestern Ontario, Canada located 40 miles (65 kms) southwest of Toronto, Canada's most populous city. With a population of roughly 160,000, the city is served by one acute care hospital, one resident long term care hospital for complex medical, and rehabilitation services and one center for specialized psychiatric and addiction services. The study used data collected through the use of the BMHS by police officers of the Guelph Police Service, the 14th largest municipal police service in Ontario with 323 members. The site of the study was specifically chosen because, as one of the original pilot sites for the BMHS, its officers have had extensive experience using the instrument and there were several years of data that could be analyzed.

### Data Collection Instrument

The BMHS is a short, 23-item, evidence-informed mental health screening assessment instrument currently used by over 40 police services in Canada and the USA (29). Developed through an international effort, the BMHS is used to help police officers identify persons with the greatest probability of falling into a high risk category based on indicators of risk of harm and disordered thought. A primary source of items on the BMHS is the RAI-MH, the mental health assessment system currently used across the province of Ontario whenever a patient is admitted into psychiatric care. The BMHS standardizes observations made by police officers and acts as a framework to enable officers to better articulate the grounds to believe a person has a serious mental disorder. Given the terminology used on the form is consistent with the RAI-MH, the language itself acts as common currency between the systems promoting better integration of systems and services.

Guelph city police officers received initial training on how to complete the BMHS, followed by additional training on how to use the accompanying software. They were instructed to complete the assessment on any member of the public who in the course of their duties, they had reason to believe had a mental health disorder. A software system (29) was built around the BMHS enabling police officers to digitally complete the BMHS assessment on a terminal located in their patrol cars at the scene of the incident. The software also calculates three clinically validated algorithms embedded in the BMHS for risk of harm to others, harm to self, and self-care (the criteria for police apprehension). Risk scores are tracked over subsequent interactions with police officers providing objective clinical feedback to help police officers decide on the most appropriate course of action. The risk scores do not dictate action but rather support a police officer's decision and can be used to justify a police apprehension when communicating with hospital staff. After completing the assessment, the police officer wirelessly transmits a summary of observations made at the scene of the incident to predetermined destinations which could include a hospital and any designated community mental health agencies. In this way, if the decision is made to transport the person to the ED, the hospital receives an early notification of incoming patients. If the assessment is sent to community agencies, it provides the information and consent (captured by the officer on scene) to initiate a follow up and provide counseling or support services if deemed appropriate.

### Data

The software allows for digitized records of assessments to be securely stored in a databased at the Guelph police station and according to a research agreement with interRAI, all users of the BMHS must agree to forward de-identified data to the interRAI Canada data repository at the University of Waterloo where the investigators had access.

### Analysis

The purpose of this analysis was to calculate cost savings over time that result from reductions in ED visits after implementation of the BMHS. To do this, using data collected through the BMHS, the total time of calls in minutes is compared between calls that resulted in an involuntary transport to the ED and the total time of calls that did not result in a transport to the ED. Next, the cost of the additional time associated with involuntary transports to the ED is calculated by using the hourly cost of police time (based on wages and benefits = $54/h) and multiplying that cost by the additional number of minutes and the number of police officers that were involved with the call. For example, if there were two officers involved with the call and the involuntary trip to the ED added an extra hour to the call, the additional cost would be $108 (1 h × 2 officers × $54). This calculation is done for all 3 years combined (2018-2020). The total wait time in the ED is also calculated for each individual year to assess whether implementation of the BMHS reduces ED wait times from year to year.

Next, the proportion of all mental health related calls that resulted in (1) an involuntary transport to the ED; (2) a voluntary transport to the ED; and (3) no transport to the ED is calculated. As with the cost analysis, this calculation is done for all 3 years combined as well as for each of the 3 years after implementation of the BMHS. Additionally, the percent change in the proportion of mental health calls with each of these three outcomes from 2018 to 2020 is calculated.

## Results

Typical of the majority of police services, data collected by the service is almost exclusively crime-related which is used as a basis for estimating police budgets. Police services therefore, generally do not collect data specific to non-crime related incidents and in particular, calls for service identified as being related to mental health. References to mental health calls will appear in a police database but will be vastly under-estimated because most references to mental health issues are subsumed under the crime-related activity that is being reported. Thus, there was no existing pre-BMHS implementation data meaning that the analysis for the current study focused on changes in successive years the BMHS was used.

During the study period extending from January 18, 2018 to December 31, 2020, there were a total of 6,727 BMHS assessments (calls for service) completed by Guelph police officers (see Table 1). The total number of calls for service identified as being related to mental health increased by 28.1% over the study period from 1,898 in 2018 to 2,425 in 2020. A total of 1,280 (19.0%) involved a trip to the ED while 5,447 (81%) did not result in an ED referral. Referrals to the ED decreased 31.2% from 481 (25.4%) in 2018 to 331 (13.6%) in 2020 while the number of incidents where there were no ED referrals increased by 48.3% from 1,412 (74.6%) in 2018 to 2,094 (76.4%) in 2020.

**Table 1:** ED referrals compared to no ED referrals by year.

**Action**	**2018**	**2019**	**2020**	**Total 2018-2020**	**% Change 2018-2020**
	***N*(%)**	***N*(%)**	***N*(%)**	***N*(%)**	
ED referrals	481 (25.4)	468 (19.4)	331 (13.6)	1,280 (19.0)	−31.2%
No ED referrals	1,412 (74.6)	1,941 (80.6)	2,094 (86.4)	5,447 (81.0)	48.3%
Total calls	1,893 (100)	2,409 (100)	2,425 (100)	6,727 (100)	28.1%

Police officer referrals to the ED were further subdivided into voluntary and involuntary. Voluntary referrals to the ED involved situations where the PSMD voluntarily agreed to accompany the police officer to the ED whereas involuntary referrals were those where a police officers used their legislative authority to apprehend and transport a PSMD to the ED for the purposes of a psychiatric examination. For the 3-year period of the study, of those persons taken to the ED, 868 (67.8%) were involuntary referrals or apprehensions and 412 (32.2%) voluntarily agreed to accompany police officers to the ED (see Table 2). Involuntary referrals decreased by 30.1% from 326 in 2018, to 228 in 2020, and voluntary referrals decreased by 33.5% from 155 in 2018 to 103 in 2020.

**Table 2:** Involuntary ED referrals compared to Voluntary referrals by year.

**ED referrals**	**2018**	**2019**	**2020**	**% Change over time 2018-2020**
	***N* (%)**	***N* (%)**	***N* (%)**	
Involuntary	326	314	228	−30.1%
	67.8	67.1	68.9	
Voluntary	155	154	103	−33.5%
	32.2	32.9	31.1	
Total	481	468	331	−31.2%
	100	100	100	

When comparing the average time police officers devoted to involuntary referrals to the ED compared to no referrals to the ED, the time spent on involuntary referrals decreased from 123 min in 2018 to 113 min in 2020 (see Table 3). There was a very slight increase in time devoted to calls where there was no trip to the ED from 80 min in 2018 to 82 min in 2020. As to the time police officers were required to remain in the ED, the average wait time for voluntary referrals dropped from 41 min in 2018 to 27 min in 2020, while involuntary referrals also saw a drop from 61 min in 2018 to 42 min in 2020.

**Table 3:** Average call times (minutes): Involuntary referrals vs. No referral.

**Action**	**2018 (SD)**	**2019 (SD)**	**2020 (SD)**
Involuntary ED referral	123 (72.1)	110 (55.9)	113 (51.9)
No ED referral	80 (81.4)	79 (72.6)	82 (70.5)

To calculate the cost of calls for service identified as being related to mental health, service calls were broken down into minutes and multiplied by the number of attending police officers and then multiplied by the salary of a first class constable which was $54.24/h or $0.90/min. Thus, the average time per call for an involuntary referral was 116 min factoring in the number of police officers at 2.6 involved which cost on average $215 per call compared to an average of 81 min where no hospital referral was involved which cost an average of $134 per call. Thus, the difference between involuntary referrals to the ED and No ED referrals was an average of 35 min at an average of $81 per call (see Table 4). In regard to the time police officers had to remain in the hospital, there was a decrease in time (minutes) over the study period for both involuntary and voluntary referrals. For involuntary referrals, police officers waited 42 min in the ED in 2020 down from 61 min in 2018 and for voluntary referrals 27 min in 2020 from 41 min in 2018 (see Table 5).

**Table 4:** Average time (minutes) and cost per call: Involuntary referral vs. No ED referral.

**Action**	**Average time/call (minutes) (SD)**	**Average cost/call (SD)**
Involuntary ED referral *N* = 868	116 (61.4)	$215 ($165)
No ED referral *N* = 5,447	81 (74.1)	$134 ($155)
Difference	35 (96)	$81 ($226)

**Table 5:** Average hospital ED Wait Times (minutes): Voluntary vs. involuntary referrals.

**Action**	**2018**	**2019**	**2020**
Involuntary ED referral	61 (52.0)	50 (48.7)	42 (24.4)
Voluntary ED referral	41 (40.7)	33 (24.8)	27 (22.5)
Total ED referrals	54 (49.5)	45 (43.2)	37 (24.8)

## Discussion

The purpose of the current study was to evaluate the costs and cost efficiency of the police response to calls for service identified as being related to mental health using the BMHS. The number of such calls for service increased by almost 30% over the study period which could be explained by the fact that police officers were increasingly more aware of indicators of mental health problems and in completing the BMHS. As to ED referrals, fewer persons were taken to the ED over the study period with an overall decrease of just over 30% and a decrease of 30% for involuntary referrals which may be due to officers having a more informed understanding of when to apprehend PSMDs. In other words, with the use of the BMHS, the police are now better able to determine when an ED visit is needed and when it is not needed. Also, further analysis would be needed to determine is the lower number of referrals to the ED is related to increased activity of the CRT which commenced activity in 2015.

An unfortunate limitation of the current study is the lack of baseline pre-implementation data that results from the fact that police services generally do not collect non-crime related data. Therefore, only decreases in ED referrals over time after implementation of the BMHS could be assessed. Because the largest decreases in ED referrals are likely to take place immediately after implementation of the BMHS, this study likely greatly understates the actual cost savings achieved by implementing the BMHS. Even so, this study still demonstrated significant reductions in ED referrals. The lower number of ED referrals may be related to the fact that identification practices may have changed over the period of observation that in turn led to fewer ED referrals and lowered costs. That is, with the implementation of the BMHS, police officers were required to complete the BMHS on all persons exhibiting indicators of mental health issues, some serious and some not as serious. The increase mental health calls could be the result of an increase in the number of BMHS assessments completed on persons exhibiting less serious indicators who in the past would have been ignored by police officers. With an increase in cases (or BMHS assessments being completed), the percentage of persons taken to the ED would naturally decrease with a corresponding decrease in associated costs. However, this assumes that increases in identification occurred from the first year of implementation of the BMHS onward, as increased identification from pre-BMHS to the first year of implementation cannot be assessed because pre-BMHS data on MH visits is not available.

Another limitation was the inability to determine from the data the proportion of persons taken to the ED who were subsequently admitted into psychiatric care. A higher proportion of admissions could support the argument that the BMHS enables police officers to identify the most appropriate persons to be taken to the ED which should result in a decrease in overall time officers are involved in ED referrals and less time spent in the ED, both of which should lead to cost-savings. Finally, a more general limitation of the study is the lack of a control group which would may have identified other factors influencing the results such as changes within the police service, community, or world (e.g., COVID-19) could have impacted or caused the findings.

An additional benefit of using the BMHS, is its ability to collect such statistics that police leaders can use to justify their budgets. This is the first study that examined specific costs associated with the use of a novel strategy to improve the police response to calls for service identified as being related to mental health. This analysis of the costs savings associated with the use of the BMHS helps to demonstrate that implementing the BMHS is a worthwhile investment for police services As was noted in a recent study involving the BMHS, the successful implementation of any form of new technology is problematic, and requires training and familiarity with the instrument (43). However, the BMHS shows great promise in predicting which calls evidence potentially serious mental health issues, and may require admission at the ED. As more and more police services in Canada and now the US implement the BMHS, and as the instrument continues to be developed and refined, it is anticipated that police officers, hospitals and community agencies will become increasingly familiar and comfortable in using the BMHS to support decision making, with the goal of improving outcomes. Although this analysis demonstrates that cost savings are achieved with continued use of the BMHS, the actual cost savings accrued from implementation of the BMHS are undoubtedly larger. Future studies should attempt to devise some method of collecting pre-implementation data so that the true cost savings of using the BMHS can be calculated. Police services could also consider the use of the BMHS in conjunction with the other models discussed in this paper as it may be possible to more accurately evaluate their effectiveness and cost-effectiveness with more accurate data pertaining to the number of calls for service related to mental health.

## Data Availability Statement

The datasets presented in this article are not readily available because the dataset analyzed for this study can be found in the Canadian repository for interRAI Canada at the University of Waterloo, Waterloo, Ontario, Canada. Only interRAI Fellows have access to the data. Requests to access the datasets should be directed to https://uwaterloo.ca/interrai-canada/.

## Ethics Statement

The studies involving human participants were reviewed and approved by Nipissing University Research Ethics Board. Written informed consent for participation was not required for this study in accordance with the national legislation and the institutional requirements.

## Author Contributions

RH coordinated the project and wrote the draft of the manuscript and along with GB formulated the research questions. JH and HK were responsible for the data analysis, creating the charts and tables, and assisting in framing the research questions. All authors approved the submitted version of the work.

## Conflict of Interest

The authors declare that the research was conducted in the absence of any commercial or financial relationships that could be construed as a potential conflict of interest.

## Publisher's Note

All claims expressed in this article are solely those of the authors and do not necessarily represent those of their affiliated organizations, or those of the publisher, the editors and the reviewers. Any product that may be evaluated in this article, or claim that may be made by its manufacturer, is not guaranteed or endorsed by the publisher.
